# New Perspectives on How to Discover Drugs from Herbal Medicines: CAM's Outstanding Contribution to Modern Therapeutics

**DOI:** 10.1155/2013/627375

**Published:** 2013-03-24

**Authors:** Si-Yuan Pan, Shu-Feng Zhou, Si-Hua Gao, Zhi-Ling Yu, Shuo-Feng Zhang, Min-Ke Tang, Jian-Ning Sun, Dik-Lung Ma, Yi-Fan Han, Wang-Fun Fong, Kam-Ming Ko

**Affiliations:** ^1^School of Chinese Medicine, Beijing University of Chinese Medicine, Beijing 100102, China; ^2^College of Pharmacy,University of South Florida, Tampa, FL 33612, USA; ^3^School of basic medicine, Beijing University of Chinese Medicine, Beijing 100102, China; ^4^School of Chinese Medicine, Hong Kong Baptist University, Hong Kong; ^5^Department of Chemistry, Hong Kong Baptist University, Hong Kong; ^6^Department of Applied Biology & Chemical Technology, Hong Kong Polytechnic University, Hong Kong; ^7^Division of Life Science, Hong Kong University of Science & Technology, Hong Kong

## Abstract

With tens of thousands of plant species on earth, we are endowed with an enormous wealth of medicinal remedies from Mother Nature. Natural products and their derivatives represent more than 50% of all the drugs in modern therapeutics. Because of the low success rate and huge capital investment need, the research and development of conventional drugs are very costly and difficult. Over the past few decades, researchers have focused on drug discovery from herbal medicines or botanical sources, an important group of complementary and alternative medicine (CAM) therapy. With a long history of herbal usage for the clinical management of a variety of diseases in indigenous cultures, the success rate of developing a new drug from herbal medicinal preparations should, in theory, be higher than that from chemical synthesis. While the endeavor for drug discovery from herbal medicines is “experience driven,” the search for a therapeutically useful synthetic drug, like “looking for a needle in a haystack,” is a daunting task. In this paper, we first illustrated various approaches of drug discovery from herbal medicines. Typical examples of successful drug discovery from botanical sources were given. In addition, problems in drug discovery from herbal medicines were described and possible solutions were proposed. The prospect of drug discovery from herbal medicines in the postgenomic era was made with the provision of future directions in this area of drug development.

## 1. Introduction

**Figure 1:**
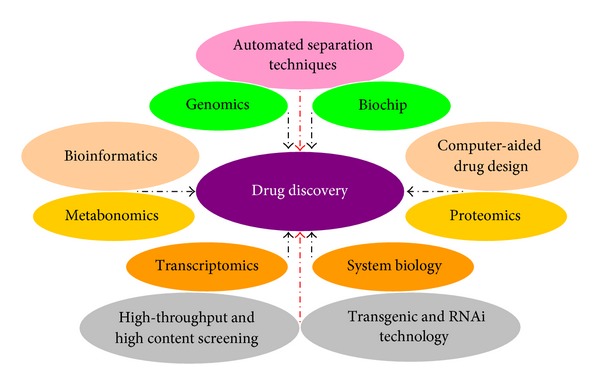
An array of technology platforms driving drug discovery.

The pharmaceutical industry is one of the pillar industries for economic development worldwide. In 2006, global spending on prescription drugs topped US$ 643 billion and the USA accounted for almost half of the global pharmaceutical market, with US$ 289 billion in annual sales [1]. Nowadays, humans cannot live well without medicine, particularly in the developed countries. Although drug discovery has been driven by a variety of technology platforms (Figure 1), which can also expedite the development of therapeutic agents from herbal medicines, drug development remains a lengthy process with a low rate of success and huge capital investment. On average, it takes about 10–15 years for a newly synthesized compound to become a marketable therapeutic agent, and the cost in 2006 was approximately *€*1 billion [2]. In 2005, Big Pharma spent about US$ 50 billion, which is more than double the amount spent in 1996. However, in that year only 22 new drugs were approved by the Food and Drug Administration (FDA) in USA, whereas 53 new drugs reached the market in 1996 [3, 4]. Despite the protracted time course of development, only one or two in ten thousand of such chemical compounds have proven to be clinically efficacious and safe for approval by regulatory agencies. In fact, about half of all drug candidates fail in the late stages of clinical trials. Furthermore, soon after their approval, some new drugs have to be withdrawn from the market due to severe side effects and clinical risks that are not detected in Phase III trials. For example, Vioxx (rofecoxib), which was launched in 1999, was withdrawn in 2004 due to an increased risk of heart attack in users. As a result, the drug only existed in the market for 5 years, and this was compounded by the huge sum (US$ 4.85 billion) of compensation involved in lawsuits [5]. Given that the development of synthetic chemicals for therapeutic use is, by and large, a random process that might result in serendipitous discovery, many pharmaceutical companies are now focused on the development of plant-derived drugs.

Complementary and alternative medicine therapies (CAMTs), which have been gaining popularity throughout the world, are classified into drug-based CAMT and nondrug-based CAMT [6]. Among various drug-based CAMTs, medicines (more than 80%) commonly practiced are herbs of plant origin. Human beings have evolved from the simple herbivorous animals to omnivorous ones. As such, we now eat both plants and meats. A wealth of experience on the application of herbal/plant materials used in promoting health has accumulated over centuries, and the information is readily available for modern scientific research on drug discovery. The earliest evidence of human use of plants for healing can date back to the Neanderthal period [7]. As a consequence, plant-derived herbs may interact favorably with the human body and hence produce beneficial effects in terms of health promotion. Interestingly, it has been observed that nonhuman vertebrates will eat some types of plants for self-medication under disease conditions [8–10]. Not surprisingly, the observations of eating behavior and serendipitous events in wild animals have led to the discovery of herbs/plants with therapeutic potential. In this regard, the study on self-medication in animals may offer a novel approach to drug discovery for humans. For instance, novel antimalarial compounds were isolated from the leaves of *Trichilia rubescens* based on a behavioral survey of chimpanzees from a natural population in Uganda [2].

In two recent articles, we have discussed a total of ten approaches in new drug discovery as well as the research and development of traditional herbal medicine “(THM)” in relation to modern drug discovery [11, 12]. While advances in new technologies have revolutionized the process of drug discovery, the successful development of a novel therapeutic agent is critically dependent on whether the process pays respect to nature, adopts new approaches/concepts, and involves the studies on ADMET (i.e., absorption, distribution, metabolism, excretion, and toxicity) in the early stages of drug discovery. Furthermore, the safe and optimal use of herbal medicines requires a full understanding of their ADME and herb-synthetic/herb-herb interaction profiles [13–16]. In this paper, we focused on the discovery of chemicals for therapeutic usage from herbal medicines, especially THM with an extremely valuable, rich, lengthy, and extensive practical history.

## 2. Current Approaches of Drug Discovery from Herbal Medicine 

**Figure 2:**
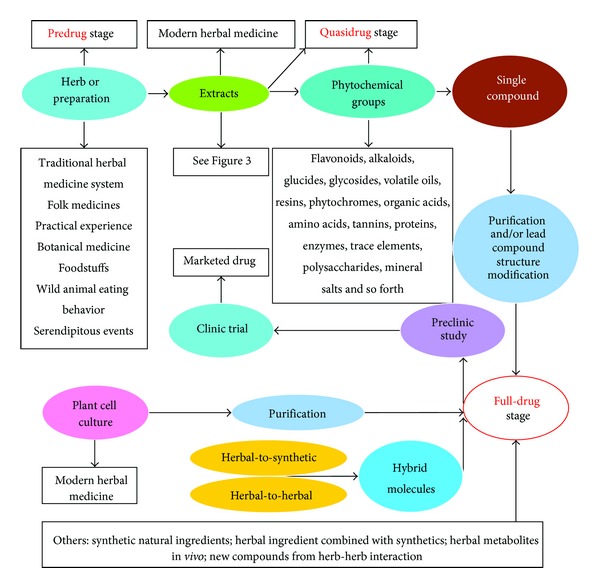
Current approaches for drug discovery from herbal medicines.

Today, approximately 80% of antimicrobial, cardiovascular, immunosuppressive, and anticancer drugs are of plant origin; their sales exceeded US$ 65 billion in 2003 [17]. It is widely accepted that more than 80% of drug substances are either directly derived from natural products or developed from a natural compound [18]. And, in fact, around 50% of pharmaceuticals are derived from compounds first identified or isolated from herbs/plants, including organisms, animals, and insects, as active ingredients [19]. Drug discovery from herbs may be divided into three stages, namely, predrug stage, quasidrug stage, and full-drug stage. They are described in detail in Figure 2.

### 2.1. Predrug Stage

It has been estimated that approximately 420,000 plant species exist on earth, but for most of these only very limited knowledge is available. Three approaches, which are closely related to diet (foodstuffs), medical practice (folk and traditional medicines), and scientific research (phytochemical analysis), can be adopted to explore the value of herbal preparations. Based on the experience from random trials and observations in animals, ancient people acquired the knowledge of using herbs for treating illness. In this connection, Chinese herbal medicine (CHM) and Indian herbal medicine (IHM), which were highly developed in ancient China, Japan, Korea, and India, are still influencing the modern healthcare [20]. The World Health Organization (WHO) estimates that herbal medicines provide primary healthcare for approximately 3.5 to 4 billion people worldwide, and about 85% of traditional medicine involves the use of plant extracts [21], which may be called “modern herbal medicine.” 

Plants, which constitute a major component of foodstuffs in humans, have formed the basis of various traditional medicine systems and folk medicines that have been practiced for thousands of years during the course of human history. Until now, plants/herbs are still highly esteemed all over the world as a rich source of therapeutic agents for the treatment and prevention of diseases and ailments; at present, more than 35,000 plant species are used for medicinal purposes around the world [22]. In conventional Western medicine, 50–60% of pharmaceutical commodities contain natural products or are synthesized from them; 10–25% of all prescription drugs contain one or more ingredients derived from plants [23]. 

It is well known that the medicinal value of herbs/plants depends on the presence of biological active ingredient(s) with drug-like properties. Recent research has identified a lot of biologically active substances/ingredients from both terrestrial and marine botanicals. For example, by 2007, 3, 563 extracts and 5,000 single compounds from 3,000 THMs have been collected in China [24]; the United States has screened about 114,000 extracts from an estimated 35,000 plant samples against a number of tumor systems as early as before the 1990s [25]. The search for new drug from plant/herb has been rapidly increasing in recent few decades, and it has led to the collection of a remarkably diverse array of over 139,000 natural products [26]. All these compounds are potential candidates for drug development. During the period 1981 to 2006, 47.1% of a total of 155 clinically approved anticancer drugs were derived from nature in North America, Europe, and Japan market [27].

The practice of traditional or folk medicine relies heavily on the use of plant-derived herbs, which can be categorized into two major types: (1) herbs which occur in databases that provide detail and coherent description of the history and theory of use, for example, herbs used in traditional Chinese medicine (TCM, *Zhong-Yi* in Chinese) and Ayurvedic medicine, and (2) herbs used in folk medicine which lack literature information on their history and theory of use. In general, herbs with a long history and theory of use in clinical settings should be more promising candidates for drug discovery. The development of modern medicine still draws on clinical experiences from traditional medicines and therapies. In China, at least 130–140 new drugs, either single chemical entities extracted from herbal medicines or synthetically modified compounds, are currently in clinical use [11]. Some examples are anisodamine [28], indirubin [29], huperzine [30], and bicyclol [31].

Traditional knowledge and experiential databases derived from clinical practice are instrumental in increasing the success rate of drug discovery by reducing the time consumed, money spent, and toxicity occurrence—which are the three main hurdles in drug development, when compared with the conventional approach adopting random screening and chemical synthesis. For example, goldthread (*Coptis chinensis* Franch), a herb being used in TCM and Ayurvedic medicine for the treatment of inflammatory symptoms and various infectious disorders for more than 3,000 years [32], was found to contain berberine—a substance that possesses powerful antibiotic and anti-inflammatory properties [33, 34]. Without the information on the use of goldthread in traditional medicines, it would have been impossible to uncover the antimicrobial and anti-inflammatory activities of berberine by random screening. More importantly, berberine has been found to produce other pharmacological actions that may have implications in clinical conditions such as diabetes, cancer, depression, hypertension, and hypercholesterolemia, all of which have been documented in the literature on goldthread [35]. Rauwolfia alkaloids, psoralens, holarrhena alkaloids, guggulsterons (an herbal supplement made from the sap of the *Guggul*), mucuna pruriens, piperidines, bacosides, picrosides, phyllanthins, curcumines, withanolides, and many other steroidal lactones and glycosides have provided fruitful outcomes in the area of drug discovery drawing on Ayurvedic experiential databases.

Herbs used in traditional or folk medicines constitute only a small portion of naturally occurring plants. With the advances in analytical technology and biological science, many bioactive chemical entities have been identified in plants or foodstuffs through phytochemical and pharmacological studies. For example, taxol (paclitaxel), an important anticancer drug, is isolated from the Pacific Yew tree [36]. Lutein isolated from marigold is known to positively affect visual performance and help prevent cataracts [37]. Lycopene from tomatoes is thought to prevent certain types of cancers [38]. While humans have mastered the technology of drug synthesis, plants remain a good source for drug discovery. As such, many popular drugs, such as paclitaxel, vincristine, vinblastine, artemisinin, camptothecin, and podophyllotoxin, were all derived from plants and developed by pharmaceutical companies. 

To sum up, “predrug stage” encompasses the information-driven selection of herbs or plants in the first stage of drug discovery from herbs/plants.

### 2.2. Quasidrug Stage

The “quasidrug” stage in drug discovery from herbal medicine includes the preparation of extracts and phytochemical groups from herbs, including the discovery of lead compounds by using modern and conventional research tools. Phytochemical study of extracts of herbal preparations or botanicals involves isolation, structure/composition elucidation, and bioactivity evaluation [39]. Sometimes, the biological activity of an herb or herbal extract can be speculated on basis of the phytochemical composition. Plant polysaccharides, which are polymers consisting of either mono- or disaccharides joined together by glycosidic bonds, produce stimulating/suppressing effect on immune system [40], and the immunomodulatory polysaccharide-containing herbs include *Ganoderma lucidum* (Leyss.ex Fr.) *Karst.* [41], *Cordyceps sinensis* (Berk.) *Sacc.* [41], and *Açaí fruit* [42]. Flavonoids, which are compounds with a heterocyclic ring structure consisting of an aromatic ring and a benzopyran ring with a phenyl substituent and include flavones, isoflavone, flavonols, flavonones, and xanthones, have been shown to possess strong antioxidant, anti-inflammatory, antiproliferative, and antiaging activities [43, 44]. They are found in almost all plants, and some of them may be used for ameliorating cardiovascular, mood problems, and cancer [45, 46]. Amino acids and proteins in herbs are usually regarded as natural nutritional supplements for patients recovering from diseases [47].

**Figure 3:**
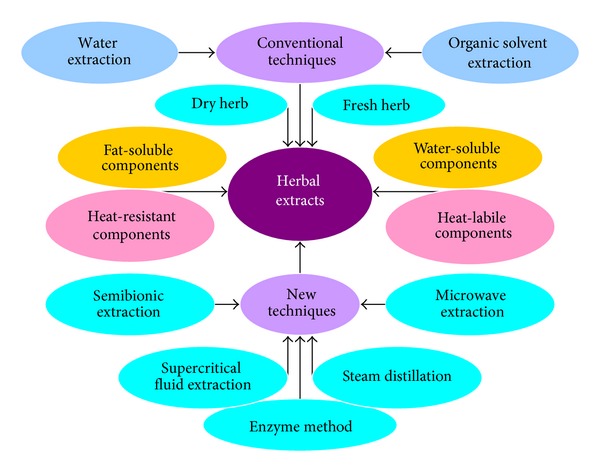
Current extraction techniques for herbal medicines.

Following harvesting, portions of the herb/plant that are used as herbs (leaves, bark, roots, flowers, seeds, or fruits) are dried either in air or using a specialized industrial drier. The dried herbs can be cut or ground into finer particles and then extracted with either water or organic solvents at an herb to solvent ratio of 1 : 4 (w/v). If fresh herbs are used, the herb to solvent ratio is 1 : 1. Physical (lipid soluble versus water soluble) and chemical (heat resistant versus heat labile) properties of the ingredients present in herbs should be taken into consideration when carrying out the extraction process. Water and organic solvent extraction is the standard technique in the pharmaceutical industry for the isolation of bioactive components from materials [48, 49]. Steam distillation is used in the manufacture and extraction of essential oils from botanical materials. The hot steam forces open the pockets in which the oils are kept in the plant material and then the volatile oils escape from them and evaporate into the steam [50]. The applications of several new technologies—supercritical carbon dioxide extraction technology, membrane separation technology, semibionic extraction method, molecular distillation technology, and enzyme method in extracting effective components of medicinal plants such as THM—have been induced in the pharmaceutical industry [51–54].  Figure 3 shows the current methods of herbal extraction.

In traditional medicines, herbal remedies are mainly made of a decoction from a mixture of raw herbs, and this dosage form is still widely used in China, Japan, and Korea. The disadvantages of herbal decoction include (1) inconvenience in terms of usage, (2) unstable in composition, and (3) issues related to quality control. The deficiencies of herbal decoctions can be overcome by using herbal extracts. Plant/herbal extracts, including those derived from THMs, have become a popular dosage form of phytomedicine and/or health products in the global market. In the USA, while health products based on plant/herbal extracts constitute more than 95% of market share in herbals, raw herbs comprise less than 5% [55].

Herbs contain hundreds of active ingredients that may be potentially useful for the development of therapeutic agents. Identification and isolation of phytochemical groups and/or single chemical entities from herbs or plant materials are therefore crucial for drug discovery. It has been estimated that there are at least 15 major phytochemical groups in herbs (Figure 2); each group contains many individual chemical entities. For instance, flavones include more than 9,000 known structures [56]. Alkaloids are an important class of active ingredient in herbs/plants, and more than 10,000 alkaloids have been isolated [57], of which more than 80 compounds have been clinically used, including berberine from *Coptis chinensis *Franch. for bacterial infection and inflammatory disorders [32, 33], ephedrine from *Ephedra sinica* Stapf. for asthma [58], reserpine from *Rauvolfia verticillata* (Lour.) Baill. previously used to treat hypertension but no longer used [59], camptothecin from *Camptotheca acuminate*, and vincristine from *Catharanthus roseus* for cancers [60, 61]. In addition, the plant secretions such as resins with potential application as antimicrobial agents can also be a source of therapeutic drugs including haemostatic, antidiarrhetic, antiulcer, antimicrobial, antiviral, wound healing, antitumor, anti-inflammatory, and antioxidant [62].

The same family or genus of plants often contains the same or similar chemical components. For example, *belladonna*, Solanaceae, and *Datura* genus contain the same substance, scopolamine. The same compound can also be distributed in different families so that berberine is not only found in berberidaceae, but also in ranunculaceae and rutaceae plants. Saponins from *Panax ginseng* can also be found in *Panax notoginseng* (Burk.) F.H. Chen, another THM distributed throughout the southwest of China, Burma, and Nepal [63]. The same bioactive ingredients may be present in various plant parts; for example, ginseng leaf stem contains numerous active ingredients (ginsenosides, polysaccharides, triterpenoids, flavonoids, volatile oils, polyacetylenic alcohols, peptides, amino acids, and fatty acids), and they also present in the root [64, 65]. The same plant species can often contain different kinds of compounds of the same class; for example, there are 25 and 70 kinds of alkaloids identified in raw opium and vinca, respectively [66].

In summary, the aim of the quasidrug stage of drug discovery from herbal medicines is to search for an active herbal ingredient or lead compound from herbs or plant materials for further drug development. It is well known that the prodrug design is an extremely challenging task for chemists in the synthesis of potential therapeutic agents.

### 2.3. Full-Drug Stage

Nowadays, drugs have become a daily necessity for many people, especially the elderly with multiple health problems. In China, for example, there are 187,518 kinds of home-manufactured drugs, 8,492 kinds of imported drugs, and 1,489 patent-protected products of THM (Chinese patent medicine, *Zhong-Cheng-Yao* in Chinese) in the pharmaceutical market, and 8,409 drug candidates are now undergoing clinical trials [67]. In addition, in the 2005 Chinese Pharmacopoeia, 582 herbal medicines are officially recognized and described. In fact, there are about 13,000 herbs and over 130,000 prescriptions currently used in various traditional medicines in China [68]. In USA, 2,900 chemical entities are currently under research and development, including agents to be used in the treatment of cancer (750), cardiovascular diseases (312), diabetes (150), AIDS (109), and Alzheimer's/senile dementia (91) [69]. 

Multinational pharmaceutical companies typically spend an annual amount of US$ 110 billion in an attempt to discover new drugs from herbal medicine. In 2003, the 10 largest pharmaceutical companies spent US$ 0.54 billion on research and development of THM, and the American AIDS Prevention Center conducted studies on screening more than 300 kinds of herb for anti-AIDS activity. In 2002, Novartis announced that 500 compounds derived from THMs would enter the patent application process [70]. At present, the development of herbal medicines mainly remains at the quasidrug stage in China (i.e., to develop modern herbal medicine) while multinational pharmaceutical companies are keen to exploit the novel drug entities (therapeutic agents) from herbal or plant extracts. In this regard, current approaches in the development of herb-derived chemicals for therapeutic use are described in detail in our previous review articles [11, 12]. 

In particular, pharmaceutical chemists are interested in hybrid molecules consisting of two distinct drug entities covalently linked in a single molecule [71], such as the hybrid of M_1_ muscarinic receptor agonist xanomeline and the cholinesterase inhibitor tacrine [72]. The hybrid molecule may contain natural-to-natural, such as tanshinol-borneol ester [73], natural-to-synthetic, such as HA'(10)-tacrine [74], or synthetic-to-synthetic components. Protein-protein hybrid may also be possible in bioharmceuticals [75]. Because of the high potential of naturally occurring compounds in producing pronounced biological activities, they have become a major source of components used for constructing hybrid molecules in the development of anticancer agents, antioxidants, and antimalarial drugs [76]. Since ancient time, multicomponent herbal formulae have been adopted in the herbal medicines. For example, there are mixtures of tea, known as jamu, containing 30 different kinds of plant species in Indonesia [77]. Over three-quarters of sicknesses are normally treated with jamu in this country. In this regard, the herb-herb interaction in multicomponent herbal formulae may create novel chemical entity such as hybrid molecule(s). 

The direct approach in drug discovery from herbal medicines is to isolate active ingredient(s) from the respective herbs or plant source. Whether or not this approach is feasible mainly depends on (1) the concentration of the bioactive component(s) in the herb or plant, (2) the degree of difficulty in purification, and (3) the availability of the herb or plant; in particular whether the plant is an endangered species.

## 3. Typical Examples of Drug Discovery from Herbal Medicine 

Antimalarial drugs developed from *Artemisia annua* (*Qing-Hao* in Chinese) and therapeutic agents for the treatment of hepatitis developed from the fruit of *Fructus schisandrae chinensis* (FSC) (*Wu-Wei-Zi* in Chinese) are the typical examples of successful drug discovery from herbal medicines. These remarkable achievements in research and development of THM have received worldwide acceptance. 

### 3.1. Antimalarial Drugs

Malaria, which is currently the most prevalent and devastating infectious diseases, is a mosquito-borne infectious disease of humans caused by eukaryotic protists of the genus *Plasmodium*. It affects nearly 40% of the global population and approximately 3 billion people in 109 countries [78, 79]. Malaria causes more than one million deaths worldwide each year; over 90% of them occur in Africa [80]. Cerebral malaria has a mortality rate as high as 20%, which can rise to 50% in pregnancy, and severe malarial anemia can have a mortality rate of over 13% [81]. Surviving patients with cerebral malaria, with more than 575,000 cases annually (children in sub-Saharan Africa are the most affected), have an increased risk of neurological and cognitive deficits, behavioral difficulties, and epilepsy [82]. Currently, chemotherapy remains the mainstay of interventional strategy in malaria control.

Archaeological findings from Mawangdui Han Dynasty Tombs from 200 BC indicated that the herb *Qing-Hao* derived from *Artemisia annua* has been used in herbal remedies for “lingering heat in joints and bones” and “exhaustion due to heat/fevers” in China for over two thousand years. The use of *Qing-Hao* for treating malaria dates back to the fourth century [83]. *Ge Hong*, a famous physician in TCM, described a herbal prescription against “intermittent fevers” (malaria) in one of his written works. In essence, fresh plant materials of *Qing-Hao* were soaked in cold water and then squeezed for juice for oral intake [83]. *Qing-Hao* is native to temperate Asia, including Korea, Japan, Vietnam (north), Myanmar, India (north), and Nepal. Interestingly, there are five kinds of *Qing-Hao* in nature, namely, *Artemisia apiacea* (*香蒿*), *Artemisia parviflora* (*小花蒿*), *Artemisia capillaris* (*茵陈蒿*), *Artemisia japonica* (*牡蒿*), and *Artemisia annua* (*黄花蒿*). However, only *Artemisia annua* contains artemisinin, an active antimalarial ingredient [84]. 

**Figure 4:**
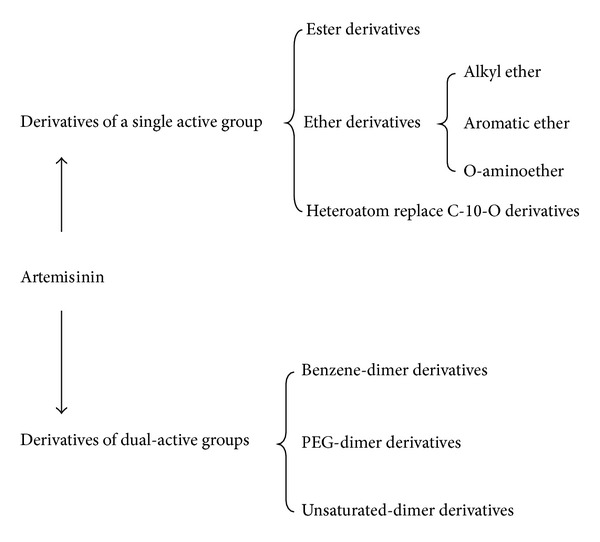


Artemisinin, also known as *Qinghaosu* in Chinese, is a highly oxygenated sesquiterpene. Unlike most other antimalarials, artemisinin contains a unique 1,2,4-trioxane ring structure, which is responsible for its antimalarial activity [85, 86]. Naturally occurring artemisinin is insoluble in water and oil, with poor oral bioavailability (8–10%) that limits its effectiveness [87]. Reduction of the lactone in dihydroartemisinin and artemether has led to increased oil solubility, whereas the acidic moiety lends water solubility to artesunate [88, 89]. Consequently, artemisinin derivatives, such as dihydroartemisinin, artemether, and artesunate, are developed and being used around the world as effective antimalarial drugs, including those targeted against multidrug-resistant *Plasmodium falciparum* [90]. These derivatives appear to be more potent than the parent compound and the most rapidly acting among all other antimalarial agents [91]. Molecular approaches in optimizing artemisinin derivatives are summarized in Figure 4 [92].

**Figure 5:**
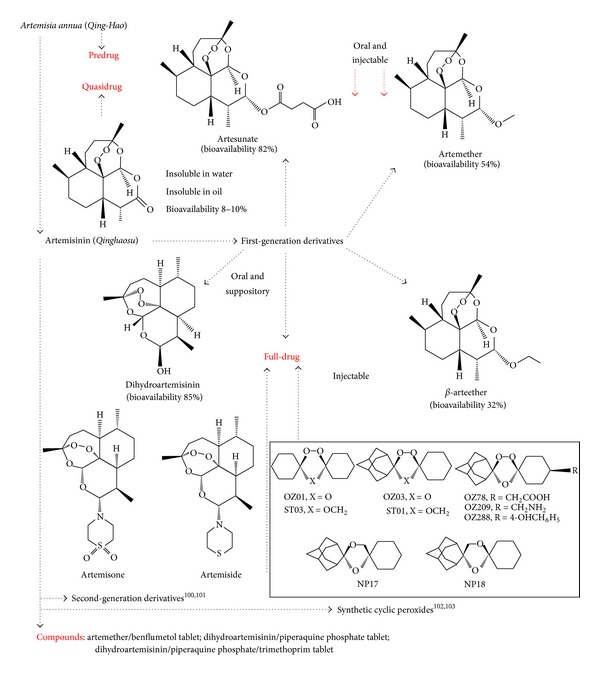
Antimalarial drugs derived from *Artemisia annua*, a Chinese herb.

The bioavailabilities following oral administration of dihydroartemisinin, artesunate, and artemether are 85, 82, and 54%, respectively [87]. *β*-Artemether, an oil-soluble ethyl ether derivative of dihydroartemisinin, consists primarily of three substituted ring systems fused together [93]. Second-generation semisynthetic artemisinin derivatives and fully synthetic antimalarial endoperoxide drugs have been developed [94–96]. The research on *Qing-Hao* not only involves the development of antimalarial drugs (see Figure 5), but also explores its potential use in anticancer, antiangiogenesis, antiviral, immunosuppressive, and antifungal therapy [97].

### 3.2. Liver Protective Agents

Hepatitis, an inflammatory disease of the liver, is commonly caused by drug intoxication or viral infection, with the latter being classified into types A, B, C, D, and E. According to WHO, 2 billion people worldwide have been infected by the hepatitis B virus (HBV), and among them 350–400 million are chronic HBV carriers, and about 1 million deaths are caused by HBV infection every year [98]. It is estimated that around 1.2 million and 3.2 million people in the USA are battling chronic hepatitis B and C, respectively, and more than 85,000 new cases of hepatitis are diagnosed each year [99]. In China, there are 93 million HBV carriers, and among them 30 million are patients with chronic hepatitis B [100].

Bicyclol, which was approved in 2001 as a therapeutic agent for hepatitis in China, has obtained patent protection in 15 countries and regions [101]. The development of bicyclol highlights drug discovery from THM. FSC, Chinese magnolia vine fruit or orange magnolia vine fruit in English, is a commonly used THM. According to TCM theory, FSC possesses five kinds of flavors, namely, pungent, sweet, sour, bitter, and salty, which bespeak much of its theraputic potential. Two kinds of *Wu-Wei-Zi*, *Bei-Wu-Wei-Zi* (*Schisandra chinensis* (Turcz.)) and *Nan-Wu-Wei-Zi* (*Schisandra sphenanthera* Rehd. et Wils.) were first recorded in *Shen-Nong-Ben-Cao-Jing* (Shennong Emperor's Classic of Materia Medica) compiled in the first century AD. Only the former is widely used in clinical practice as an herb with a broad profile of therapeutic actions, including antitussive, antiasthmatic, sedative, and antihepatitis and tonic actions [102–104].

In the 1970s, it was found that FSC, in a dosage form of powder or bolus, could decrease serum alanine aminotransferase (ALT) activity and improve symptoms in patients suffering from viral hepatitis. Experimental investigations also demonstrated the hepatoprotection afforded by FSC against chemical toxicant-induced injury in rodents [105]. Activity-guided fractionation of the ethanol extract of FSC resulted in the isolation and identification of a series of active dibenzocyclooctadiene derivatives that could suppress serum ALT activity in animal models of hepatitis [106, 107]. Their relative potencies in serum ALT suppressive activity are in a descending order: schizandrer B > schizandrol B > schisandrin C > schisandrin B > schisandrin A > schizandrer A > schizandrol A [108]. Schisandrin C, which has an intermediate potency among other dibenzocyclooctadiene derivatives in lowering serum ALT, was selected for drug development. Because the synthesis of schisandrin C was difficult [109], bifendate, a synthetic intermediate of schisandrin C, was chosen for further investigation as a drug candidate [110–112]. 

**Figure 6:**
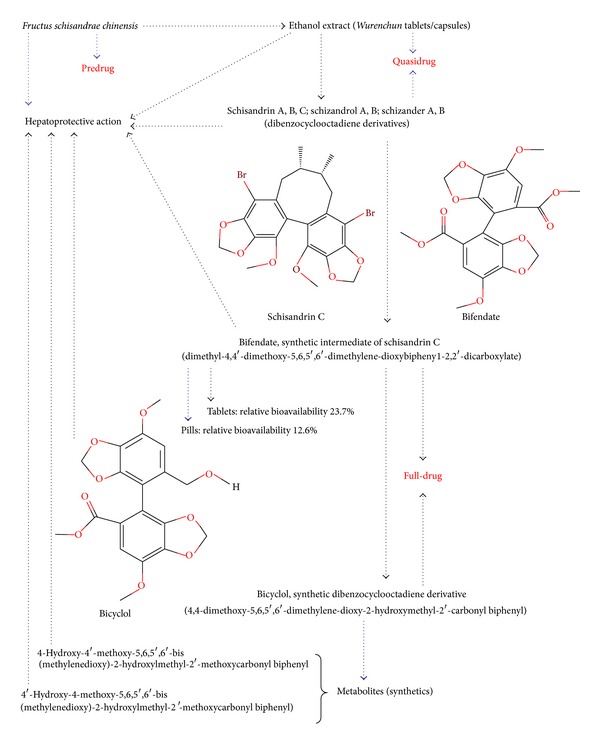
The discovery of therapeutic agents for hepatitis from *Fructus Schisandrae chinensis* (FSC), a Chinese herb.

**Figure 7:**
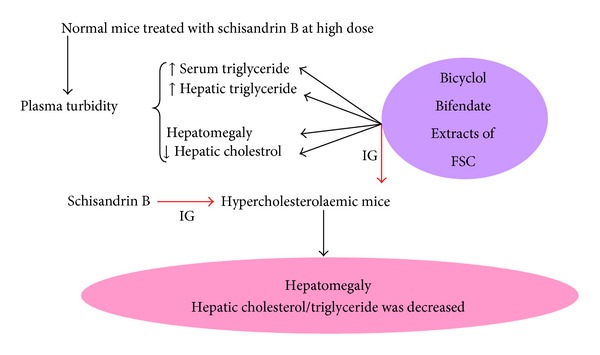
The discovery of *Fructus schisandrae Chinensis* (FSC) for modulating lipid metabolism.

Bicyclol, a second-generation synthetic antihepatitis drug on the market, is synthesized based on bifendate. It has been shown to be more potent than bifendate in protecting against liver injury caused by toxicants or viral infection and preventing liver fibrosis [113–115]. Furthermore, it has been found that the hepatoprotective action of (−)-bicyclol was two times more potent than that of the racemic bicyclol, and the potency of (+)-bicyclol was much less than that of the racemate [116]. In addition, two metabolites of bicyclol *in vivo* were identified and chemically synthesized, but their hepatoprotective activity was lower than that of bicyclol [117]. The drug development process of FSC is summarized in Figure 6. In addition, it has recently been found in our laboratory that FSC and its related synthetic compounds can affect the lipid metabolism including an increase in serum and hepatic triglyceride levels as well as liver weight in normal mice, but a decrease in liver lipids in hypercholesterolaemic mice (see Figure 7) [118–125]. Schisandrin B inhibits FFA-induced steatosis in L-02 cells by, at least in part, reversing the upregulation of ADRP and SREBP-1 [126]. These findings thus open a new avenue for FSC research.

## 4. Systems Pharmacology and Drug Discovery from Herbal Medicine

**Figure 8:**
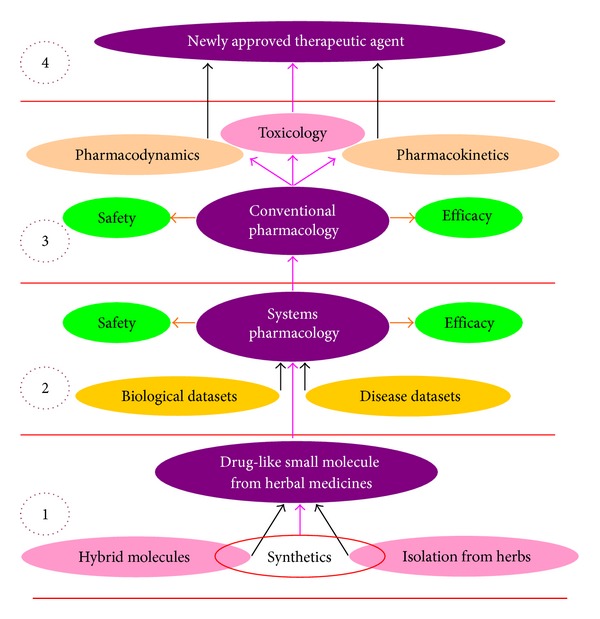
Current processes in drug discovery from herbal medicines.

The typical drug development process from herbal medicines includes at least four differential aspects: (1) isolation or artificial synthesis of bioactive ingredient(s) in herbal medicines; (2) evaluation of safety and efficacy using systems pharmacological methods; (3) evaluation of safety and efficacy by means of conventional pharmacological methods; and (4) regulatory approval of the therapeutic agent to be used in the market and postmarket monitoring. This concept is illustrated in Figure 8. Here, the impacts of systems pharmacology on drug discovery from herbal medicines are discussed.

### 4.1. Database in Network

Systems pharmacology, a new emerging field, uses both experiments and computational analysis of regulatory networks to develop new understanding of drug action across multiple scales of complexity ranging from molecular and cellular levels to tissue and organism levels [127]. It enables drug discovery from both natural remedies and synthetics and makes predictions about the possible therapeutic effects and adverse events, including possible off-target effects, through combination of the structural analyses with analysis of regulatory networks [128, 129]. It seems to be clear that systems pharmacology provides new approaches for drug discovery for complex diseases such as cancer, diabetes, and heart diseases. Over the past decade, genomic, proteomic and metabolomic technologies, network analysis, and other high-throughput studies, as well as structural and biochemical studies, have provided a wealth of information for integrated systems-level analysis of drug action and molecular targets, including the beneficial and adverse effects for humans [130, 131]. 

It is well known that drugs are designed to treat specific pathophysiological conditions by interacting with specific target(s) and cause a change of either excitement (positive respective) or suppression (negative response). It may be recorded as the initial insight on the molecular mechanisms of drug action. After understanding the structure of compounds from herbal medicines, one can estimate their potential pharmaceutical value and adverse effects via systems pharmacological analysis. 

Potential Drug Target Database (PDTD), a comprehensive and web-accessible database of drug targets, has collected 1,193 protein entries with 3D structure covering 831 known and potential drug targets in the Protein Data Bank (PDB) [132–135]. Drug targets of PDTD were categorized into 15 and 13 types according to two criteria: therapeutic areas and biochemical criteria [132]. By 2000, only about 500 drug targets had been reported [136, 137], among which only 120 drug targets are actually marketed [138]. Until 2009, however, PubChem, a public repository of small molecules and their biological properties, contains over 1,700 biochemical and cell-based bioassay screens, nearly 60 million biological activity outcomes for different protein and gene targets, which provide biological annotations for more than 750,000 unique small molecule chemical structures and tens of thousands of siRNA probes. By 2010, the BioAssay database is comprised of more than 2,700 bioassays associated with more than one million compounds tested against several thousand molecular targets [139]. Currently, it contains more than 25 million unique chemical structures and 90 million bioactivity outcomes associated with several thousand macromolecular targets [139]. 

About 3,700 unique entries in the Online Mendelian Inheritance in Man (OMIM), which is a comprehensive, authoritative, and timely compendium of human genes and genetic phenotypes, constitute the diseasome. The database also covers a tiny fraction of the proteome consisting of approximately 100,000 proteins [128]. World of Molecular BioAcTivity (WOMBAT) [140], a leading small molecule chemogenomics database and the standard of quality in small molecule bioactivity annotations, contains 322,638 entries (263,187 unique SMILES), totaling over 798,595,700 biological activities on 1,966 unique targets so far [140, 141]. A bipartite interaction network has been constructed using 1,284 known disorders and 1,177 known disease genes from the OMIM database [128]. There are more than 14,000 targets on the DrugBank database website [142, 143]. This database contains 6,796 drugs including 1,437 FDA-approved small molecule drugs, 134 FDA-approved biotech (protein/peptide) drugs, 83 nutraceuticals, and 5,174 experimental/investigational drugs.

**Figure 9:**
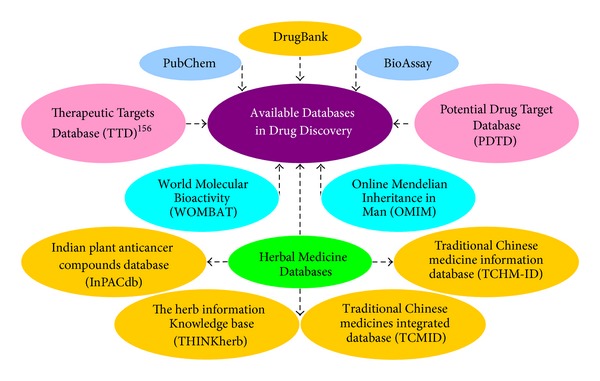
Available databases in drug discovery.

Currently, herbal medicines are applied widely and researchers have growing interest in scientific proof and molecular analysis of herbal effects. Therefore, several herbal medicine databases containing abundant scientific data underlying the use and study of herbs for health have been adopted for using in the Internet. For example, the herb information knowledge base (THINKherb) database, an innovative herb database to provide integrated and knowledge-based information, contains 499 herbs, 1,238 genes involving human, mouse, and rat, 825 diseases, 245 pharmacological activity, and 373 signaling pathways (human 148, mouse 121, and rat 104) [144]. Traditional Chinese medicine information database (TCM-ID) currently contains information for 1,588 prescriptions, 1,313 herbs, 5,669 herbal ingredients, and the 3D structure of 3,725 herbal ingredients [145]. The value of the data in TCM-ID was illustrated by using some of the data for an *in silico* study of molecular mechanism of the therapeutic effects of herbal ingredients and for developing a computer program to validate herbal medicine multiherb preparations [146]. Traditional Chinese medicine integrated database (TCMID) records ∼47,000 prescriptions, 8,159 herbs, 25,210 compounds, 6,828 drugs, 3,791 diseases, and 17,521 related targets [147]. TCHMID displays a network for integrative relationships between herbs and their treated diseases as well as the active ingredients and their targets. Indian Plant Anticancer Compounds Database (InPACdb) is unique in providing comprehensive information on phytochemicals of Indian origin with anticancer activity via 32 descriptive fields [148, 149].  Figure 9 shows these databases that will help us to find new drugs from herbal medicines.

The completion of human genome and numerous pathology-related genomes suggests that many of the 30,000 to 40,000 identified proteins are potential targets for drug discovery [150]. It has been estimated that there are more than 2,000 potential drug targets with at least one drug candidate in clinical trial [150, 151]. At present, these databases have become a valuable resource for drug development and attracted considerable interest from researchers in both academia and industry. As to how these known targets can be utilized to screen drug candidates derived from herbal medicines for disease treatment as well as to discover the new use of an old drug present a challenge for pharmaceutical industries and biotechnology companies. Currently, the synthetic drug is designed with reference to the three-dimensional structure of disease and/or biological targets presented in various databases. Conversely, a drug-like small molecule from herbal medicines is usually regarded as ligand for screening related target(s) from both biological and disease data sets. Further research will be guided by the information obtained through systems pharmacology. Undoubtedly, systems pharmacology will greatly expedite the development of therapeutics, particularly for complex diseases. 

### 4.2. Problematics

Despite the fact that systems pharmacology offers a powerful new approach for drug discovery in the preclinical phase, the research and development (R&D) effort required for a drug to proceed from clinical trials to market involves a large amount of time and money [152]. This might be due to the following reasons.

Firstly, at present, although a lot of disease/drug targets including about 3,700 genes/proteins associated with diseases have been identified, only a few drug targets are successfully used in current therapies [153], and about two-thirds of drugs that are currently used in the world do not target a gene product that has been associated with a disease. Nearly 50% of drugs fail in Phase III trials due to the lack of efficacy or demonstrated benefit as compared to a placebo or an active control or as an add-on therapy. There are more than 1,500 drugs approved by the FDA, but only approximately 400 unique proteins are targeted by these approved drugs [128]. Many biomarkers, also known as targets, are proposed in highly cited studies as determinants of disease risk, prognosis, or response to treatment, but only a few of them are eventually transformed into clinical practice [154].

Secondly, single and/or multitargets cannot represent the individual and the mechanism of drug action at the whole body or *in vivo* condition. Currently, for example, the large amount of information amassed over the years from biochemical and cell-biological studies has indicated that single-target/gene disorders are rare, and most diseases are associated with multiple genes, and some diseases are associated with 30 to 50 genes or more than 100 genes. Most genetic diseases are caused by the concerted action of many target and/or genetic abnormalities, and they include metabolic syndrome [155, 156], cancers [157], hypertension [158], and mental illness like Major Depressive Disorder [159], Post-Traumatic Stress Disorder [160], schizophrenia [161], AD [162], and inflammatory/infectious diseases like arthritis [163] and AIDS [164]. Furthermore, a disease target may have many family members. Compounds cannot simultaneously affect all disease-associated targets at gene, protein, and other biomarker levels. Therefore “magic-bullet” therapy to guide the treatment of disease and drug development has not demonstrated a high success rate, even though antisense drugs (referred to as “magic bullet”) are used for the treatment of various conditions such as diabetes, hypertension, and autoimmune and cardiovascular diseases [165, 166].

Thirdly, complex diseases have multiple disease and/or therapeutic targets including genes, enzymes, ion channels, transporters, receptors, binding protein, structural proteins, signaling proteins, factors, regulators, and hormones, along with other possible nontargeting factors such as environmental, cultural, social, educational, and climatic factors, hormone levels, age, weight, and lifestyle factors including diet. These nontargeting elements may control and/or modify the function and structure of disease/drug targets even gene expression *in vivo*. These determinant factors dictate disease manifestation and drug effects/toxicity, including off-target effects. Unfortunately, this point is often overlooked in medicinal science studies. Here, stroke is regarded as an example to illustrate the importance of nontarget factors in the disease and drug effects. Approximately 500,000–600,000 Americans suffer stroke every year, which is the third leading cause of death and number one cause of permanent disability in the USA [167]. Therefore, the research and development of therapeutic agents for stroke management have attracted much attention. So far, many stroke-related and therapeutic targets have been identified at various levels, as well as over 500 treatment strategies in animal models of stroke including gene therapy as a novel pharmaceutical intervention [168, 169]. However, if animals are cooled (<35°C), infarct volume was reduced by about one-third and the outcomes were improved in animal model of stroke [170]. On the contrary, hyperthermia has consistently been shown to increase infarct volume in animal models of focal cerebral ischemia [171]. These findings suggest that cool condition, which can turn off all stroke-related targets like therapeutics, has considerable potential as a neuroprotective strategy in patients with acute stroke [171, 172]. It directs to a more integrated approach for health care than the isolation of single molecules for specific targets for drugs. In addition, the enhanced longevity of the Japanese may be related to the relative late expression of the aging gene, which in turn may be related to the Japanese diet and/or lifestyle. The nontargeting factors may therefore influence the manifestation of target function. 

Finally, many drugs, particularly those derived from natural products, hit multiple targets, each of which exists within a complex network. As such, approximately 35% of known drugs or drug candidates are active against more than one target [173]. One drug molecule may interact with several targets that contribute to the overall effectiveness of a treatment, including targets associated with toxicity. For example, curcumin, a polyphenol natural product isolated from Rhizoma Curcumae Longae, at least, has ten molecular targets associated with anticancer, antiviral, antiarthritic, antiamyloid, antioxidant, and anti-inflammatory properties [174]. Nexavar, an orally active multikinase inhibitor, was originally designed to inhibit Raf kinase isoforms for treating renal and liver cancers, but it was also found to inhibit PDGF and VEGF receptor tyrosine kinases [175]. Therefore, the anticancer action of Nexavar involves at least three targets, including the suppression of cancer cell proliferation by inhibiting Raf and the prevention of tumor progression and angiogenesis by inhibiting PDGFR and VEGFR. If both targets reported in the literature and predicted targets are taken into account, there are 6.3 targets per drug [176]. This can lead to multiple outcomes ascribable to both beneficial and harmful effects of the drug.

### 4.3. Some Thoughts

Current drug R&D is complemented by a growing volume of bioinformatics information on the drug candidate to ensure a high success rate in clinic trials by predicting adverse drug reactions and efficacy in the early stage of drug development. However, many drugs still failed at the Phase III stage, meaning that the drugs could not show a statistically significant improvement in efficacy including toxicity. This problematic situation indicates that new strategies are necessary for drug discovery.

By the time new drugs are made to Phase III trial, 66% of them failed due to the lack of efficacy, that is, no significant difference between the placebo and drug treatment or between drug and current treatment alternative, and 21% failed because of safety concerns [177]. The high failure rate of drugs in clinical trials indicates that a variety of drug targets make no sense in clinical trials. Those drugs that failed in clinical phase have gone through a lot of target research in preclinic phase, including animal safety and effectiveness evaluation at molecular target levels, which are published in reputable journals. 

In contrast to systems pharmacology, conventional pharmacology mainly evaluates the potential efficacies and side effects of drug candidates under experimental and clinical investigations. Systems pharmacology is a virtual science based on datasets supported by genomics, proteomics, and metabolomics, and so forth, in the network, but conventional pharmacology is a practical science based on experimental studies. Effects of drug *in vivo* are controlled by a complicated network of targets, including the biochemical and functional response (signals) resulting from the interaction of drug and target(s). Conclusions obtained by virtual science are often far from the facts; for example, about 90% of real off-target effects cannot be predicted and excluded by bioinformatics [178]. Even though the disease/drug target(s), molecular mechanism, is known, the effectiveness of the drug in clinic trial cannot be guaranteed. 

Analyzing the signaling network involved in a drug action within the context of a patient's genetic background may be critical to the success of the therapy, but it is not translated directly to effectiveness in clinical condition. Many biologically active molecules are very active *in vitro*, but never reach the clinical stage. There is always a discrepancy between theory and practice, particularly in medicinal science. For example, *β*-lactamase-producing bacteria, which can degrade *β*-lactam antibiotics and render antibiotics inactive in inhibiting bacterial cell wall synthesis, can be overcome by the combined use of *β*-lactamase inhibitors and *β*-lactam antibiotics [179]. However, an estimated number of 25,000 patients have died of bacterial infections that are resistant to antibiotic treatment every year in hospitals of the European Union region [180]. In 2005, almost 19,000 people in the USA died from an infection called methicillin-resistant *Staphylococcus aureus* (MRSA) [181]. Therefore, the effectiveness in clinical use is the only way to determine the validity of results obtained from basic study in therapeutic and medicine.

There are thousands of chemical entities in human body and most of them can either be disease or drug target, or even both. They may be considered as first-order disease/drug targets, second-order disease/drug targets, positive disease/drug targets, negative disease/drug targets, critical disease/drug targets, supplementary disease/drug targets, and nondisease/drug targets. For disease emergence and intervention, the target(s) may be either blocked or activated by disease-causing factor and therapeutic agents. In theory, once targets on disease or pathogen are identified, candidate drugs can be obtained by selecting existing drugs or by designing the candidate drug at the molecular level with a computer-aided design program. Although more and more diseases and pathogen targets are revealed, the success rate of new drug R&D has become lower than ever. The treatment of modern disease has also become increasingly difficult, and sometimes patients have to be left helplessness. While the identification of disease and drug target is important, the determination of interrelationship among targets is crucial for drug development. Life science is a study of interrelationships among cells, tissues, and organs. Physiological processes in human body are regulated by a complicated network of signaling pathways. Hypothetically, each physiological component of the network is represented by a node, and each interaction is represented by an edge between two nodes [182, 183]. In a disease system, the signaling network underlying the pathological symptoms is most likely perturbed at more than one point (node or edge).

Due to the increased incidence of complex and refractory diseases with multiple targets, at present, combination therapy (i.e., simultaneous administration of two or more medications to treat the same disease) based on insight obtained from genomic studies is very common. Up to February 2010, the number of clinical trials for drug combinations in USA (6,720) accounted for approximately 15% of the total number of clinical trials (44,832) [152]. The combination of subcutaneously administered pegylated interferon and oral ribavirin is the FDA-approved regimen for the treatment of chronic HCV infection [184]. Hypolipidemic drug combinations include statins with cholesterol ester protein inhibitors, niacin, fibrates or fish oil, and fibrate-ezetimibe combination [185]. Herbal products have a lot of active compounds that interact with multiple targets simultaneously. Moreover, the drug derived from herbal medicines possesses polypharmacological effects *in vivo*. For example, natural compounds for the treatment and prevention of ischemic brain injury produce multiple actions such as antioxidation, anti-inflammation, calcium antagonization, antiapoptosis, and neurofunctional regulation [186]. Because of the coevolution of humans and plants in nature, the plant-derived components of herbal medicine should have more action targets than those of synthetic drugs. This may be the background of the use of herbal medicine, multitarget approach of a disease.

## 5. Five Issues of Drug Discovery from Herbal Medicine 

**Figure 10:**
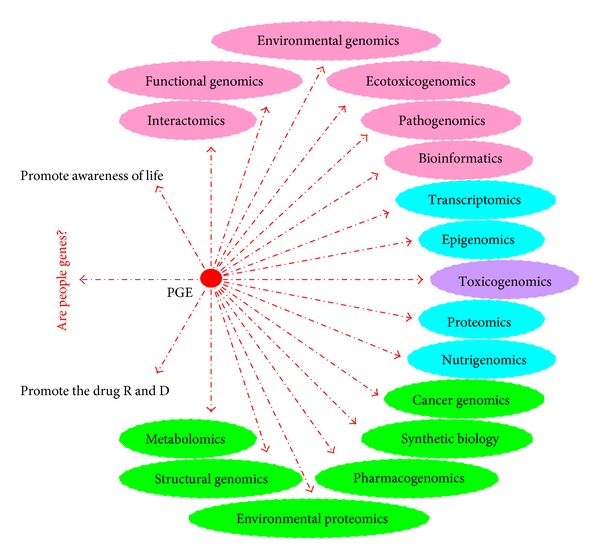
“Postgenomic” era (PGE) and the associated new disciplines of study.

On June 26, 2000, it was announced by the International Human Genome Project that a working draft of the sequence of the human genome—the genetic blueprint for a human being—was assembled [187]. Since then, human history has entered a new era—the “postgenomic” era. So far, the elucidation of the human genome has given rise to more than a dozen new related disciplines of study (see Figure 10). The impact of the “postgenomic” era on drug discovery from herbal medicine is discussed in the following.

Firstly, in this “postgenomic” era, the screening of herbal ingredients should be accelerated. Due to the development of related disciplines in the “postgenomic” era, the pace of drug discovery from herbal medicine is likely to be hastened and become more efficient than ever, with a resultant higher success rate. Unlike synthetic drugs, drugs derived from herbal medicine are developed by isolating and identifying the active chemical entities in the crude herbal extract, rather than by going through processes of drug design and synthesis. Although many aids and tools for advanced drug design are now available and the *in vivo* properties of a drug can also be predicted using quantum mechanics and statistical mechanics [188–190], the complexity of biological systems often defies some of the basic assumptions of rational drug design. As such, it is impossible to estimate all drug-related parameters in an accurate manner during the “pregenomic” era. It is estimated that a single herbal preparation may contain more than 100 active compounds [191], and their bioactivities and/or drug-like properties are difficult to be accurately identified and evaluated by conventional pharmacological methods or drug screening approaches. In addition, the levels of these compounds are exceedingly low and usually insufficient for conducting *in vivo* studies. From 1960 to 1982, 114,000 crude extracts from 35,000 plant samples were processed, but only two compounds, taxol and camptothecin, were developed into marketable therapeutics [192]. 

If the endeavor had been undertaken in the “postgenomic” era, the success rate would likely have been much higher. By performing a comparative study on gene expression profiles before and after taking the herbal extract, one can readily screen out new drug targets. Furthermore, genes responsible for regulating the pharmacodynamics and pharmacokinetics of the drugs can better be understood. As a drug can cause a differential gene expression, making use of gene profiling in drug screening is an efficient way to identify the active ingredient(s) in herbal extracts. In this regard, a research team at the Hong Kong University of Science and Technology (Hong Kong) was able to identify 23 active ingredients in *Anemarrhena asphodeloides* Bge. (*Zhi-Mu* in Chinese) using gene chip technology [193], which might not have been accomplished easily by conventional/traditional screening technologies. 

Secondly, the “postgenomic” era should lead to the identification of more drug targets. It is well known that any drug has its own *in vivo* target(s), which may be involved in the development of a disease. The Human Genome Project has identified 32 billion base pairs in the genome, including about 30,000 to 40,000 protein-coding genes in 23 pairs of chromosomes. However, the results of structural genomics research can only reveal genomic structure and sequence information, which are only the starting point for understanding biological function. During the “postgenomic” era, scientists will be able to create the human proteome (an inventory of all human proteins) and study the physiological function of the proteins. The discovery of disease-associated molecular targets at the level of DNA, RNA, protein, enzyme, or receptor has made the mechanism- and structure-based drug discovery process obsolete. Once a molecular target of disease is revealed, one can use it for identifying active ingredient(s) from herbal medicine in drug discovery. In addition, the generation of transgenic and gene knockout animals by biotechnological techniques can provide human disease models for screening drugs of clinical interest; for example, *APOE∗3Leiden.CEPT* transgenic mice have been used for screening drugs for the treatment of hyperlipidemia [194].

More often than not, the etiology of a disease is multifactorial. Commonly occurring diseases such as hypertension, cardiovascular diseases, asthma, rheumatoid arthritis, osteoporosis, and neuropsychiatric disorders are polygenic in nature. For example, Alzheimer's disease (AD) has been characterized as a complex multifactorial disorder and ten AD-related genes have so far been identified [195–197]. Huperzine A, which is a drug used in the treatment of AD patients, was originally discovered from a THM, *Huperzia serrate* [198–201]. In addition to acetylcholinesterase inhibition, it has been shown that huperzine A also scavenges hydrogen peroxide and ameliorates beta-amyloid-induced oxidative stress and glutamate-, ischemia-, or staurosporine-induced cytotoxicity/cell apoptosis, as well as inflammation, all of which are beneficial for the management of AD [198, 200–202]. With the discovery of new disease-related genes and proteins, new drug targets will become available for drug discovery from herbal medicine.

Thirdly, the “postgenomic” era will expand the herbal resources. While scientists are deciphering genes in humans and other animals, they can also do the same on plants of interest. In China, projects on genome sequencing of 100 animals/plants are in progress, of which nearly half of the projects have been completed or are near completion [203]. On June 20, 2010, Chinese scientists announced that 92% of the genome and 96% of the gene coding regions of *Salvia miltiorrhiza* Bge. had been elucidated [204]. The understanding of genomic characteristics of medicinal plants is instrumental to generate a new variety of medicinal plant with improved quality and efficacy. 

The limited supply and low concentrations of bioactive ingredients in medicinal plants have limited their usage in health management. During the “postgenomic” era, scientists may obtain the desirable compounds from transgenic plants or microbes for therapeutic applications. As such, the cost of drug development would be reduced by the application of genetic engineering. For instance, China has established an Agrobacterium-mediated transformation system for *Artemisia annua* and successfully transferred a number of genes related to artemisinin biosynthesis into the plant [205, 206]. The genetically modified plant is capable of producing artemisinic acid, an artemisinin precursor, at a significantly higher yield than from its parent plant [207, 208]. However, transgenic plants or microbes are a very costly procedure and not without safety risks.

Fourthly, a genome is not equivalent to a human body. The 21st century is regarded as the century of life science in relation to genes. Even though 99.99% of the genes among humans are the same [209], individual differences exist in various aspects of drug action, metabolic transformation, side effects, and so forth. We, therefore, believe that humans are not merely genes, or in other words genes are not equivalent to humans. DNA, RNA, or proteins are not necessarily the target in all drugs and diseases. We investigate the target(s) of drug action at the gene and/or protein levels, but we cannot guarantee that this drug can cure the disease or be efficacious for all patients. Over the last 50 years, more than 500,000 chemicals, both synthetic and naturally occurring, have been screened for antitumor activity [210]. Why, then, does cancer still represent such a major therapeutic challenge? In addition to the incomplete understanding of cancer development, it is possible that some compounds with antitumor activity resulted from the experiments yield false-positive observations during the early stages of drug discovery.

According to various genome projects, both mice and humans have about 30,000 genes; yet only 300 are unique to either organism; that is, about 99 percent of genes in humans have their counterparts in mice [211]. About 60% or more genes are conserved between flies and humans, and humans have more genes than fruit flies (approximately 30,000 compared to 13,000) [212]. If genetic exchange is allowed, will humans become mice, or vice versa? The answer to the question should be negative. After the insertion of a transposon into the red pigment gene, morning glory became white rather than the expected red color [213]. If the mouse eye gene is transferred to a fruit fly, can the fruit fly grow out of a pair of mouse eyes? Again, the answer is negative. After all, genes do not govern everything in life. Therefore, conventional pharmacological methods should also be incorporated into the process of drug discovery from herbal medicine. 

Lastly, in the “postgenomic” era, one should not abandon tradition. While we acquire more in-depth understanding of the essence of life, many advanced techniques for drug discovery have become readily available. However, it should be kept in mind that plants/herbs, which form the basis of sophisticated traditional medicines, have existed and used for thousands of years. In addition, herbs are rarely used singly in the practice of traditional medicines, but are usually used in formulae of a mixture of herbs that are prescribed according to specific medicinal principles. Moreover, herbs are processed after harvest to fulfill different requirements for therapy, dispensing, and dosage forms [214]. New ingredient(s) may be generated from herb-herb interactions or herb processing. 

The discovery of *Qinghaosu* underlines the importance of traditional knowledge in drug discovery from herbal medicine [215, 216]. At first, drug screening experiments showed that the antimalarial activity of *Artemisia annua* extracts was very poor and therefore it did not arouse scientists' attention for a long period of time. In fact, *Ge Hong* used a cold water extract of *Qing-Hao* rather than a decoction prepared with hot water for the treatment of malaria. Later, scientists modified the extraction method for *Qing-Hao* by using ethanol or ether at low temperatures. A complete inhibition of mouse malaria *P. berghei* by the *Qing-Hao* extract derived from low-temperature process was observed. Artemisinin, a novel antimalarial drug which is heat labile, was finally discovered in China. The success rate of drug development from plants based on random screening is undeniably low. The story of artemisinin has emphasized the importance of traditional information in achieving a higher success rate in drug discovery from herbal medicine.

## 6. Problems of Drug Discovery from Herbal Medicine

Strategies of drug discovery from nature resources are greatly different from those of synthetics. It involves two key issues, for example, ecological ethics and resource-dependent sustainable development.

**Figure 11:**
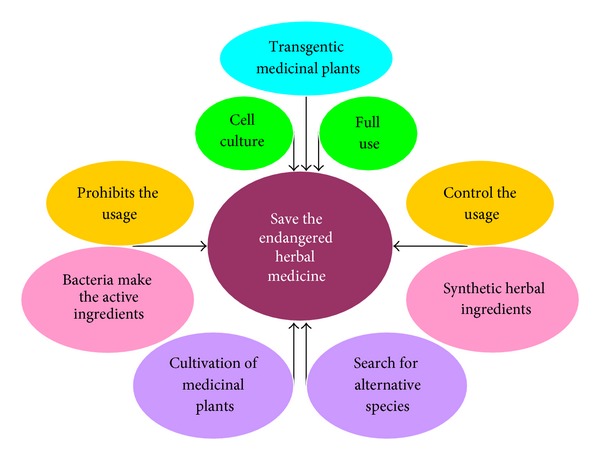
Available approaches in preserving the endangered plants or herbs used in herbal medicines.

1. The current development of herbal medicines involves both modern ethics and technologies. In this section, ecological ethics on the drug discovery from herbal medicine are discussed. Ecological ethics are moral principles governing human attitudes towards the environment and rules of conduct for care and preservation of the environment [217]. On our planet, no species, including humans, can evolve independently of its coevolution with other species. By the same token, no species can live independently of other species. As the ecological environment on earth deteriorates, the number of existing species has drastically decreased over the last five decades, with the exception of the rapidly growing humans. In fact, the imbalance between humans and other species has become a threat to the survival of humans, thereby ecosystem or plant ecology should be firstly considered in the herbal development.
2. Plants including medicinal plants have incredible biological diversity, showing extreme flexibility in ecomorphology. It is undoubtedly that plants play an important role in ecosystems by providing essential services. Without plants there would be no human, animals, and insects, in other words, no life as we understand it on earth without plants. In any case vegetation always acts as indicator of general ecosystem health. Unfortunately, to date, many species have disappeared even before they could be identified. Ten years ago, biologists warned that 25% of all species could become extinct over the next 20 to 30 years [218]. Over the past two decades, pharmaceutical companies have shown an increased interest in exploring new compounds from plants for drug development. However, the lack of a rational approach in many aspects of this undertaking, including drug discovery from herbal medicine, has limited the success of these endeavors. In 2004, the World Wildlife Fund pointed out that due to the overexploitation of human consumption, 20% of medicinal plants in the world are facing extinction. These include popular medicinal plants such as *Cypripedium pubescens*, *Trillium erectum*, *Aletris farinose*, *Chamaelirium luteum *syn. *Helonias lutea (dioica)*, *Hydrastis Canadensis*, and *Panax quinquefolius* [219], as well as many plants used in traditional herbal medicine. There are about 250,000 higher plant species on Earth and over 80,000 have good medicinal values. The past experience told us that once a plant species had been identified for its commercial potential, it may face a risk of extinction.
3. In China, for instance, there are about thousand endangered plants, of which 60–70% are medicinal plants [220]. There are nearly 12,000 species of medicinal plants used in TCM, and more than 6,000 species have disappeared over a period of less than 20 years. At present, out of 400 kinds of commonly used herbs, the supply of more than 20% of them has become depleted from natural sources [221]. For example, the annual sales of *Isatis tinctoria *L., *Ban-Lan-Gen* in Chinese, were increased from 3,000 tons in 1970s to 60,000 tons during the period from 2006 to 2008 [221]; the over consumption of *Ban-Lan-Gen* has therefore depleted its supply from natural source. Therefore, the preservation of endangered and rare medicinal plants has been an area of immense interest, particularly in China [222, 223]. Due to the market-driven overexploitation of herbal or plant resources in recent years, many herbs have been listed (or may soon be listed) as endangered species. China, where herbal preparations account for 30–50% of the total medicinal consumption, has a number of herbs listed in the China Plant Red Data Book, in which the category of “Rare and Endangered Plants” includes *Acanthopanax senticosus*, *Fritillaria ussuriensis*, *Aquilaria sinensis*, *Fritillaria walujewii*, *Astragalus membranaceus*, *Gastrodia elata*, *A. membranaceus. *var.* mongolicus*, *Ginkgo biloba*, *Changium smyrnioides*, *Glehnia littoralis*, *Cistanche deserticola*, *Illicium difengpi*, *Coptis teeta*, *Juglans regia*, *Coptis chinensis*, *Magnolia officinalis*, *Dalbergia odorifera M. o. *var.* biloba*, *Dendrobium candidum*, *Morinda officinalis*, *Dimocarpus longan*, *Panax ginseng*, *Eucommia ulmoides*, *Phellodendron amurense*, *Ferula sinkiangensis*, *Picrorhiza scrophulariiflora*, *Fritillaria pallidiflora*, and *Rosa rugosa* [224]. Many herbs from Arabian herbal medicine, including *Anchusa negevensis*, * Anchusa ovata*, *Eryngium barrelieri*, *Eryngium maritimum*, *Eryngium maritimum*, *Euphorbia hirsute*, *Ophioglossum lusitanicum*, *Ophioglossum polyphyllum*, *Teucrium procerum*, *Teucrium scordium*, and *Teucrium scordium*, have also been designated rare or endangered species [225].
4. Drug development from herbal medicine should take into account the diversity of species and ecological ethics. Ideally, during the drug development, one first isolates and identifies target compounds from herbs or plants, which is followed by total chemical synthesis of the compound(s), as was the case for aspirin. When a bioactive compound has been isolated, modification of the chemical structure may be performed in order to reduce the toxicity and increase the efficacy and solubility of the drug candidate. Acetylsalicylic acid, also called aspirin, is an acetyl derivative of salicylic acid, which is derived from the metabolism of salicin, a naturally occurring compound found in the bark of Willow and Spiraea plant 150 years ago. However, commercially available salicylic acid and aspirin are synthetics [226, 227]. Interestingly, aspirin causes a lesser degree of digestive upset than salicylic acid. Semisynthetic derivatives of artemisinin isolated from the plant *Artemisia annua*, such as dihydroartemisinin, artemether, and artesunate, were found to be more bioavailable and have greater activity than artemisinin itself [228, 229]. However, it is well known that the modification of the chemical structure of compounds derived from herbs does not always yield results with positive impact on the societies such as heroin (diamorphine) and “*Bing-Du*” (methamphetamine). Some approaches for preserving rare and endangered medicinal plants are shown in Figure 11. The ethical principles should be conformed during the development and exploitation of herbal medicine. After all, humans should not fulfill their own needs at the expense of other species on earth. On the other hand, in less developed countries herbal medicine is the primary source of health care. When these herbs are seized by big Pharma, the resulting drugs will not be available to the people because of the costs. This is also an ethical issue in drug discovery from herbal medicines. Generally, the herbal medicines that are traditionally used on their value are less expensive than the new single-component drugs from them.
5. In order to meet the escalating demand for medicinal plants, herbal agriculture or farming of these plants is imperative—and the development of agrotechnology related to herbal production has become an area of intensive research. Farming medicinal plants (*Yao-Nong* in Chinese) for herb production has provided a number of jobs in China. During 2005–2010, central and local governments as well as enterprises in China invested 115.56 million RMB for the cultivation of herbs [230]. Currently, there are 250 kinds of medical plants being cultivated or grown in China, encompassing 33.3 million hectares of farmland [231]. As of 2010, there are 99 Good Agricultural Practice (GAP) sites, and 49 species of herbs are currently cultivated at these sites [232]. In Germany, 19 species commonly used in herbs have been investigated for commercial cultivation or breeding [233]. In addition, cell and bacterial cultures as well as transgenic plants are also available for supplying source materials for drug discovery from herbal medicine. For instance, *Artemisia annua* is the commercial source of artemisinin, but the concentration of artemisinin in this herb is low, ranging from 0.01 to 0.8% of the dry weight of the plant. To meet the increasing demand in the market, transgenic plants containing a higher content of artemisinin were developed [234]. Although the use of transgenic plants is a disputable means of preserving biodiversity, genetic engineering will play an important role in saving the medicinal plants which are rare, endangered, or threatened with extinction. If the precursor (mevalonic acid lactone) and elicitor (methyl jasmonate) were added to the cell cultures, the production of artemisinin was increased by about 6 times relative to control cultures [235]. In addition, *Escherichia coli* can produce terpene amorpha-4, 11-diene, a precursor of artemisinin [236].
6. Generally, drug discovery from herbs should mainly involve synthesis and cultivation of medicinal plants, not just exploiting the wild plants in nature. Furthermore, to the largest possible extent, all parts of a medicinal plant should be utilized. For example, ginsenosides, the active ingredients of Radix Ginseng, are also found in the leaves and stems of *Panax ginseng* [237, 238]; therefore, leaves are also of medicinal values like stems. We can only harvest the leaves of *Panax ginseng* and let it grow back next year.

## 7. Concluding Remarks

As ancient humans adopted a plant-based (i.e., herbivorous) diet, the body function of humans may have been primed by a large number of secondary metabolites derived from plants. Considering the extremely high cost and long time of new drug development, as well as the high drug attrition rate, an imminent task for pharmaceutical companies is to explore new ways for drug R&D. Therefore, more and more attention in the field of drug discovery has been focused on the herbal medicine. Herbal medicine as a source of new compounds for drugs is going to become a global trend in the pharmaceutical industry [239]. 

An impressive number of chemicals have been isolated either from medicinal plants or synthesized on the basis of natural lead compounds. For instance, schisandrin C present in *Schisandra chinensis* has led to the discovery and development of two potent drug derivatives, bifendate and bicyclol. Artemisinin isolated from *Artemisia annua* has generated at least ten new drugs on the market. Therefore, the use of herbal/plant medicine has been the single most successful strategy for the development of novel therapeutic agents, and this trend will be continued in the future. 

In an era of rapidly advancing science and technology, there is a tendency to ignore traditional values and knowledge, as well as traditional medicines at large. Although the “postgenomic” era offers great opportunities for screening active compounds from medicinal plants, one should be aware of traditional knowledge in an attempt to discover drugs derived from herbal medicine. Many medicinal properties of plant species were revealed from experience accumulated from a long history of use in many traditional herbal therapies. Knowledge accumulated in traditional medicine, therefore, plays an important role in enhancing the success rate of drug discovery from herbal medicine. Generally, the success rate of the synthetic route for developing new medicinal agents may be 1/10,000; however, the success rate with search for new therapeutic moieties based on medical plants used in traditional medicinal system can be as high as 1/4 or more. 

Last but not least, the principle of ecological ethics should be upheld by preserving biodiversity while exploiting natural resources for drug discovery. Man does not have the right to wipe out any species arbitrarily and mess with genes to create transgenic crops for their own benefits. People are just one of the residents on Earth. As articulated by the ancient Chinese philosopher *Lao Zi*: “Mother Nature is benevolent to all living things even a stray dog on earth.” Modern anthropocentrism with value of philosophical significance for sustainable development should be implemented in the processes of herbal medicine R&D.
